# On the Treatment of the Dental Pulp

**Published:** 1844-10-19

**Authors:** John D. White

**Affiliations:** Philadelphia, Surgeon Dentist


### ON THE TREATMENT OF THE DENTAL PULP.

BY JOHN D. WHITE, M. D., OF PHILADELPHIA, SURGEON DENTIST.

The age at which the roots of teeth are completely formed, is a subject, which, as far as I have observed, has not been settled. Yet it is as important a point in a practical point of view, as any other connected with their development. Of course, the same irregularity with regard to the completion of the formation of the roots as to age will obtain, as with the protrusion of the teeth through the gum; consequently, it will be difficult to arrive at a knowledge of the precise period at which they are completed.

A few observations by some of the profession would, I have no doubt, soon suggest something sufficiently definite to enable us to form a correct diagnosis when called upon to treat the diseased teeth of young patients. I was first impressed with the importance of the subject about five years ago; but I confess not until several ineffectual attempts at saving incompletely formed teeth, by treating the pulp, and filling them, compelled me to seek out a deeper cause than the mere mode of treatment. I examined authors, but found nothing on the subject. Whether they have considered it as a matter which would strike the mind of any one who would attempt to operate at all, as so self evident as to require no precaution or comment, I know not; but it is certain that a few hints would have saved me some trouble, and my patients some pain, in the commencement of my practice.

I know well that there are many in the profession, of longer experience and more extensive reading than myself, who have met, and still do meet with similar difficulties by not having any very definite practice on the subjcet.

I have a number of specimens of the second molares, extracted from different patients, of fourteen years of age, in which the foramina at the extremities of the fangs are from half a line to a line in diameter. If we were to calculate the time it requires for a tooth to become completely developed after it pierces the gum, it would assist us in making out a diagnosis, and this will call for an exercise of the judgment of the practitioner; as the patient is often unable to recollect the precise time at which the molares first appear. Age, therefore, would seem to be the best guide. The teeth of patients under twenty, even though they seem to be fully developed, as far as my experience goes, are more difficult of preservation than those of older patients.

When we are consulted with regard to the teeth of young patients, we find the observance of the following rules of great value; but of course they are by no means in all cases to be relied on, but they are an approximation from which the operation must deviate, according to the nature of the case. If the pulp be exposed in a first molar of either jaw, and the patient under twelve years of age, we consider it beyond the reach of successful treatment; if it be one of the second molares, and the patient under sixteen, the treatment, as a general rule, will also be unsuccessful. In consequence of the fangs of the single teeth being farther developed when they pierce the gum, than the fangs of the double teeth, they can be treated with more safety than the latter, and in a shorter time after they make their appearance. As the patient in almost all cases observes the time at which the single teeth pierce the gum, if we add say from three to four years, it will allow sufficient time for the completion of the fangs; a regard, therefore, by the operator of the times respectively at which the single teeth make their appearance, will be essential to the formation of a correct diagnosis. If the pulp be destroyed, the farther formation of the tooth will be arrested. Intense inflammation will be produced in the external membranes and jaw, by destroying the pulp outside of the incompletely formed roots; and if it be not destroyed, inflammation will extend to the external membranes which will produce the same result.

I was solicited by a lady, about three years ago, to destroy the nerve of the first inferior molar, for her daughter, who was about eight years of age. I refused, for reasons given above; but not being satisfied with my opinion, she sought advice elsewhere, and the dentist to whom she applied destroyed the nerve, as he said, and plugged the tooth; after which intense inflammation set in, the part was poulticed externally (when poultices are applied, they should invariably be applied to the gum opposite the tooth,) to produce suppuration, and, as it was extensively inflamed and swollen, it pointed outside opposite the tooth, near the base of the jaw. The parent became alarmed, and applied to me again; I removed the tooth, as a matter of course, and found the roots of it incomplete, and the foramin large enough to admit the barrel of a crow-quill.

---

To the Editor of the Medical Examiner:

SIR—I beg leave, through your columns, to inquire of your numerous readers and correspondents, whether any of them have ever tried the experiment of raising the European leech in this country, and if so, what method they have found successful?

The high price of this valuable agent, the increasing demand for them, and the great difficulty with which they can at times be obtained, especially in parts of the country remote from large cities, justifies, I think, the attempt to rear them among us.

A communication from any gentleman who has made the experiment, would, I have no doubt, be acceptable to many of your readers. MEDICUS.

*Charlottesville, Va.*, Aug. 31, 1844.

Since the reception of the above communication we have made inquiry of an intelligent leecher in this city, on the subject to which it refers, but he knew of no instance in which the attempt had been seriously made to raise any of the foreign leech. We shall be very glad to receive and communicate any information on this subject, and hope that some of our readers will be able to answer the inquiry of our correspondent.—EDITOR.

